# A novel candidate disease gene prioritization method using deep graph convolutional networks and semi-supervised learning

**DOI:** 10.1186/s12859-022-04954-x

**Published:** 2022-10-14

**Authors:** Saeid Azadifar, Ali Ahmadi

**Affiliations:** Faculty of Computer Engineering, K. N. Toosi University of Technology, Tehran, Iran

**Keywords:** Gene prioritization, Graph convolutional networks, Protein–protein interaction, Semi-supervised learning, Gene identification

## Abstract

**Background:**

Selecting and prioritizing candidate disease genes is necessary before conducting laboratory studies as identifying disease genes from a large number of candidate genes using laboratory methods, is a very costly and time-consuming task. There are many machine learning-based gene prioritization methods. These methods differ in various aspects including the feature vectors of genes, the used datasets with different structures, and the learning model. Creating a suitable feature vector for genes and an appropriate learning model on a variety of data with different and non-Euclidean structures, including graphs, as well as the lack of negative data are very important challenges of these methods. The use of graph neural networks has recently emerged in machine learning and other related fields, and they have demonstrated superior performance for a broad range of problems.

**Methods:**

In this study, a new semi-supervised learning method based on graph convolutional networks is presented using the novel constructing feature vector for each gene. In the proposed method, first, we construct three feature vectors for each gene using terms from the Gene Ontology (GO) database. Then, we train a graph convolution network on these vectors using protein–protein interaction (PPI) network data to identify disease candidate genes. Our model discovers hidden layer representations encoding in both local graph structure as well as features of nodes. This method is characterized by the simultaneous consideration of topological information of the biological network (e.g., PPI) and other sources of evidence. Finally, a validation has been done to demonstrate the efficiency of our method.

**Results:**

Several experiments are performed on 16 diseases to evaluate the proposed method's performance. The experiments demonstrate that our proposed method achieves the best results, in terms of precision, the area under the ROC curve (AUCs), and F1-score values, when compared with eight state-of-the-art network and machine learning-based disease gene prioritization methods.

**Conclusion:**

This study shows that the proposed semi-supervised learning method appropriately classifies and ranks candidate disease genes using a graph convolutional network and an innovative method to create three feature vectors for genes based on the molecular function, cellular component, and biological process terms from GO data.

## Background

The prioritization of candidate genes involves identifying and evaluating the genes to demonstrate which ones are most likely to be associated with a particular disease or phenotype. Ranking genes based on their association with disease is the most common way to determine candidate gene prioritization. A gene with a higher rank is more likely to be associated with the disease and is more likely to be investigated further, compared to one with a lower rank. Prioritizing candidate genes is crucial since these methods will allow biomedical researchers to investigate in-depth only a limited number of potentially useful genes. Therefore, candidate gene prioritization can greatly accelerate translational bioinformatics and the advancement of new therapies [1].

PPI data provided by recent high-throughput technology [2] can serve as a critical resource for candidate-gene prioritization since genes associated with a particular disease phenotype tend to be clustered in specific locations in the network of PPI [3]. However, relatively simple techniques have been applied for gene-prioritization including searching for neighboring disease genes and finding the shortest paths between candidate genes and known disease proteins.

Computational methods use different biochemical resources in different ways to calculate the association between genes and rank the candidate disease genes. A number of databases have been produced by investigators to help deal with the problem of identifying and prioritizing genes, such as the first protein structure, signal pathways, textual sources, gene ontology, gene expression, and PPI networks [4]. Due to the fact that genes (proteins) associated with a particular disease have a tendency to interact, PPI has been used as the main dataset in most methods, and it is a source of information when generating features for each gene.

To separate candidate genes from non-disease genes, machine learning strategies such as supervised learning, unsupervised learning, semi-supervised learning, and feature extraction have been used. According to a principle known as guilt by association, a gene's function is revealed by its interactions with other genes. We can estimate candidate genes' function in prioritizing genes by discovering their relation to seed genes. The functional characteristics of seed genes are discovered using machine learning methods. Then, they compute the similarity between seed genes and candidate genes for classification [5]. One of the most widely used machine learning approaches is semi-supervised learning. Using semi-supervised learning, unlabeled data is leveraged to improve performance. In many semi-supervised learning algorithms, the supervised loss function on labeled data and the unsupervised loss function on both labeled and unlabeled data are optimized simultaneously. A graph-based semi-supervised learning algorithm models the loss function as the weighted sum of the supervised loss over labeled samples and the graph Laplacian regularization term [6, 7]. The graph Laplacian regularization assumes that nearby nodes in a graph will often have the same labels. The reason why graph Laplacian regularization is effective is that it constrains the labels to be consistent with the graph structure [8].

In spite of the wide range of disease gene prioritization methods available now, computational gene prioritization methods fall into four major categories [5], including text mining methods, network-based, machine learning, and hybrid methods. Based on the published scientific literature, the text mining method identifies gene to complex disorder associations. As the knowledge sources, these methods use GO, Human Protein Reference Database (HPRD), Kyoto Encyclopedia of Genes and Genomes (KEGG), and Medical Literature Analysis and Retrieval System Online (MEDLINE). Cosine similarity, Jaccard similarity, Pearson's correlation, latent semantic analysis, information content, and document vectorization are some examples of common text mining metrics [9–14]. Text mining-based methods have disadvantages such as the inaccessibility of information because of license and privacy concerns, limitations of text processing, reduced performance caused by the syntax and semantics of the document, and redundancy from the absence of concept organization [15].

In network-based methods, biological data is represented as a network, and gene rankings are computed using graph mining techniques. It is further subdivided into the direct neighborhood such as NGP [16], ICN [17], CIPHER-HIT [18], and resnikHPO [19] which calculates the rank of every node in the network based on its association to directly connected nodes, diffusion-based such as MAXIF [20], and HDiffusion [21] that utilize both direct and indirect node interactions for ranking, and random walk methods. Some notable examples of these methods include GPEC [22], ORIENT [23], RWRHN [24], RWRM [25]. The ORIENT method enhances the RWR method by adding weights associated with interactions close to known disease genes, while any gene-protein relationship network can be used. The RWRHN method predicts and prioritizes candidates for inherited diseases from a heterogeneous network containing such diverse genes. By merging a variety of genomic networks into a multigraph, RRRM provides a data platform, which is then used for developing a random walk algorithm by computing the transition matrix using modifying step process.

In recent years, numerous novel methods based on networks have been successfully applied to prioritizing genes, which integrate different omics data to identify candidate genes [26–29]. Nevertheless, most analysis of omics data only maps genes into networks without any further investigation of network topology. Additionally, several network diffusion methods, like DawnRank [30], spread expression information across protein interaction networks by uniformly choosing neighborly nodes as their successors. Gentili et al. [31] have proposed a BRW (biological random walk) method for leveraging biological information for gene prioritization. The random walk also has some advances in other research fields. A method for predicting lncRNA-disease associations (IRWRLDA) proposed by Chen et al. [32] is one such method. By choosing a uniform probability of seed nodes associated with disease and analyzing lncRNA expression and disease semantic similarity separately, both methods contribute to enhance the initial probability of restart term. Real-world scenarios, on the other hand, are more likely to show such tendencies and to prefer selecting the neighbor to a greater degree than uniformly. It is rarely considered by the previously mentioned random walk–based methods. The topological characteristics of seedlings have not been considered in some methods, although some realize the importance of seed genes [33]. Thus, it is beneficial to utilize topological characteristics of the graph and other information regarding nodes (genes) and propagation tendency for a novel method to identify disease genes.

Machine learning techniques, including unsupervised and supervised learning, feature extraction, etc., can be used to distinguish disease genes and non-disease genes in a list of candidate genes. The concept of 'guilt by association' indicates a gene's functionality by its interaction with other genes. Gene prioritization estimates candidate gene functionality by identifying their association with seed genes. Prioritization is fundamentally connected to the concept of machine learning, which strives to learn from past behaviors and apply this knowledge in assessing the behavior of upcoming inputs. Machine learning methods identify seed genes' functional properties and use that information to compute their similarity to candidate genes for ranking. Gene prioritization can be accomplished by machine learning techniques due to their added advantages over traditional approaches. In the literature, a variety of gene prioritization models have been developed based on machine learning algorithms. Adie et al. [34] developed a gene prioritization method named PROSPECTR based on a decision tree algorithm to rank genes according to their involvement in disease. Nitsch et al. [35] proposed three approaches to score disease candidate genes using network-based methods, including kernel ridge regression, heat kernel, and Arnoldi kernel approximation. The authors modeled brain development gene expression using a support vector machine (SVM) and a feature selection method, for the classification and prioritization of autism spectrum disorder (ASD) risk genes. ProDiGe [36] formulated the disease gene prioritization as an example of the problem known as learning from positive and unlabeled examples (PU learning).

In recent years, deep learning models on graphs (e.g., graph neural networks) have appeared in machine learning and other related areas, and achieved superior performance in a variety of problems. Graph convolutional networks are a popular type of deep graph learning technique. In terms of the spatial domain (also known as the vertex domain), graph convolution can be viewed as an aggregation of node representations based on the node neighborhoods. Introducing these operations opens a window to graph convolutional networks [37]. Graph convolutional models are a form of neural network architecture that takes advantage of the graph structure and aggregates neighborhood information in a convolutional manner [38]. Graph convolutional networks have a high level of capability in learning graph structures and have been successfully used for a variety of problems and applications, including drug discovery [39] and molecular property prediction [40]. The majority of graph convolutional networks methods [41–44] consider the problem of candidate disease gene prioritization as a link prediction problem and use convolutional graph networks and graph embedding on integrated networks. The presented methods are limited by the lack of use of other data sources with a non-graph structure and inadequate features for each node.

To rank genes, hybrid approaches utilize two or more prioritization methods. In spite of hybrid methods' numerous benefits, implementing them demands high effort, complex data management, and advanced programming techniques. NCBI and other common databases, such as HPRD, MeSH, etc., can be used as data sources for hybrid methods. A few examples in this category include PrixFixe [45], ENDEAVOR [46], GeneMANIA [47], Gene Prospector [48] and DAPPLE [49], NetRanker [50], GeneTIER [51].

**Fig. 1:**
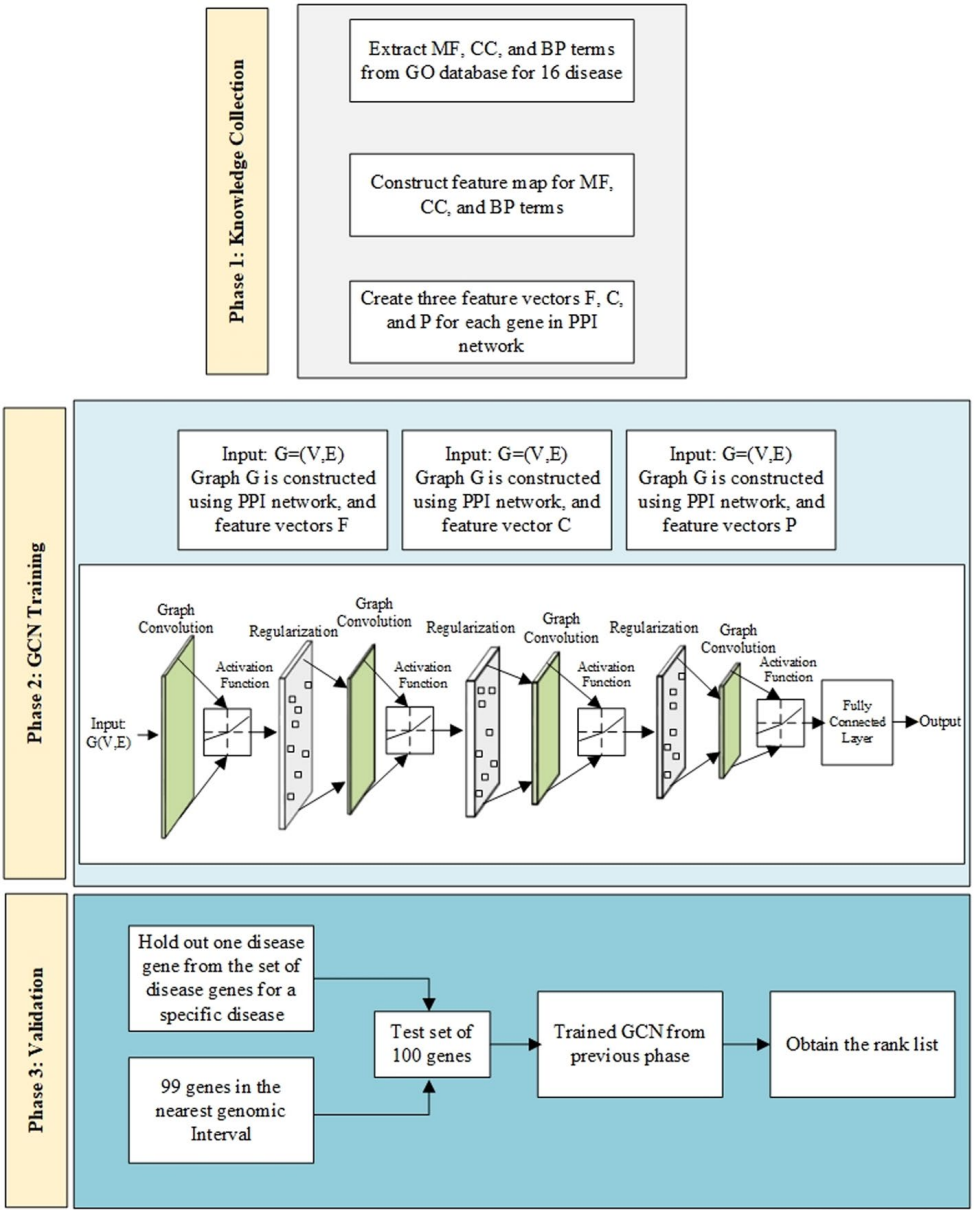
The overview of the proposed graph convolutional network for gene prioritization (GCNGP) method

There are a few limitations to the current network-based inference algorithms: they do not use node feature vectors, and they still suffer from shallow learning mechanisms. Another challenge for this problem is that there are a small portion of labeled genes and a large number of unlabeled genes, which makes conventional supervised learning techniques inapplicable. Feature vectors are a crucial component of machine learning algorithms, and a lack of them leads to reduced efficiency. In machine learning-based methods, the lack of negative data can result in negative data being picked at random, which can negatively impact their performance. The aim of this study is to provide a semi-supervised learning approach using graph convolutional networks along with an innovative method for building gene feature vectors in order to overcome the limitations of candidate gene prioritization. We consider the problem of disease gene prioritization as classifying nodes (genes) in a graph (PPI network), where labels are only available for a small subset of nodes. This problem can be framed as graph-based semi-supervised learning. The proposed method called the graph convolutional network for gene prioritization, in short GCNGP, works in three phases: First, we extract the Molecular Function (MF), Cellular Component (CC), and Biological Process (BP) terms from the GO Database for all associated genes of 16 diseases, and three feature maps are created for each one. Then, for each gene, three feature vectors are formed based on the presence or absence of the terms collected in the previous step. In the second phase, three PPI networks are created using the features derived from phase 1, and then a graph convolutional network is trained using the graphs and semi-supervised learning approach. The proposed method is validated in the third phase. In this phase, a test set of 100 genes containing a disease gene from a set of genes associated with a disease is constructed, along with 99 genes located in the nearest genomic interval. Then, these genes are ranked using the trained graph convolutional network from phase 2. We present a brief summary of the proposed model in Fig. 1. Compared to other well-known approaches, the proposed method incorporates a number of contributions:

1. For the first time, this study presents a semi-supervised learning algorithm based on graph convolutional networks that aims to prioritize disease candidate genes.
2. An innovative method based on GO data is used to construct a feature vector for each gene.
3. To rank genes, the customized convolution operator is used directly on the vertex domain, and information is propagated via the PPI network. Additionally, direct and indirect interactions between genes are considered.
4. Using both GO and PPI databases simultaneously and thereby improving the learning and accuracy of the proposed method.
5. Utilizing the structural characteristics of every node in the network along with the feature vector created for each node.

## Methods

'Guilt by association' is a principle that takes into account a gene's interaction with others in order to illustrate its functionality. Gene prioritization estimates candidate gene functionality by identifying their association with seed genes. Prioritization is fundamentally connected with the idea of machine learning, which strives to learn from past behaviors and apply this knowledge in assessing the behavior of upcoming inputs [5]. Machine learning is useful to find out how a seed gene's properties are similar to those of the candidate gene, and also to learn the ranking. During prioritizing and identifying disease genes using PPI network data, only a few genes (proteins) have a class label, and the rest are unlabeled. Therefore, we can frame this problem as semi-supervised graph-based learning [52, 53], where label information is smoothed across the graph utilizing some type of explicit graph-based regularization, such as using a graph Laplacian regularization term in the loss function [38]:

1 $${\mathcal{L}} = {\mathcal{L}}_{0} + \lambda {\mathcal{L}}_{reg} ,\;\;{\text{with}}\;\;{\mathcal{L}}_{reg} = \mathop \sum \limits_{i,j} A_{ij} \left\| {f\left( {X_{i} } \right) - f\left( {X_{j} } \right)^{2} } \right\| = f\left( X \right)^{T} \Delta f\left( X \right)$$

where ${\mathcal{L}}_{0}$ indicates the supervised loss w.r.t. based on the labeled part of the graph, $f(.)$ can be a neural network like differentiable function, $\lambda$ is a weighing factor, and $X$ represents a matrix of node feature vectors ${X}_{i}$. $\Delta =D-A$ is the unnormalized graph Laplacian of an undirected graph $G=\left(V, E\right)$ with $N$ nodes ${V}_{i}\in V$, edges $({V}_{i}, {V}_{j})\in E$, an adjacency matrix $A \in {R}^{N\times N}$(binary or weighted), and a degree matrix ${D}_{ii}=\sum_{j}{A}_{ij}$. For the formulation of Eq. (1), it is assumed that the connected nodes of the graph often have the same label.

Like the method proposed in [38], in this study, a neural network model $f(X, A)$ is used to encode the graph structure directly. Here, first, we describe how to construct feature vectors for each gene, then we discuss graph convolution, and finally, we discuss semi-supervised learning and the training model used in this study.

### Feature vector construction

**Fig. 2:**
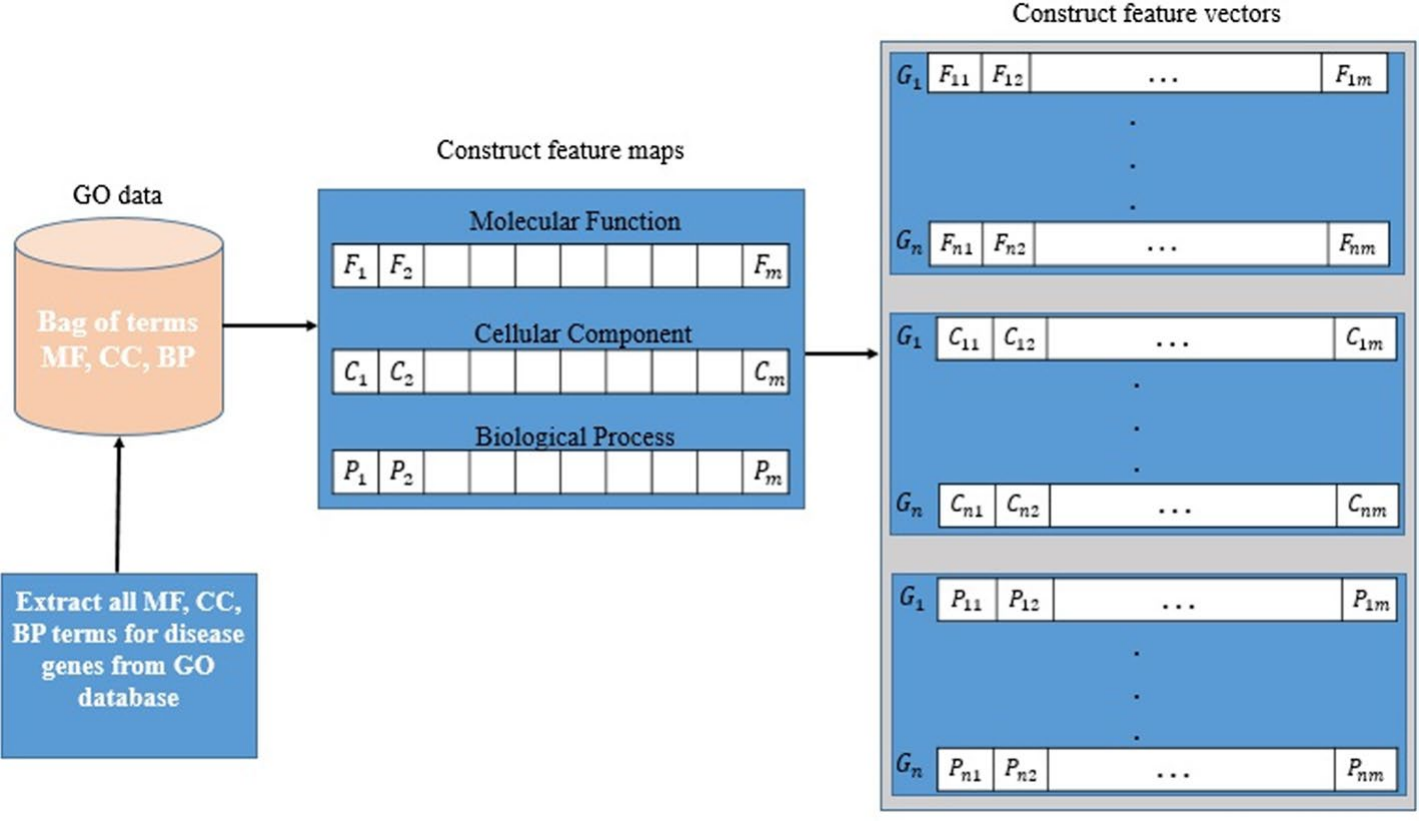
The overall schema of the proposed feature vectors construction method

The GO dataset can be viewed as a bag of terms, which contains three categories of terms. The categories include Molecular Function, Cellular Component, and Biological Process. In this paper, three feature vectors are constructed based on each category of terms using a new method. As shown in Fig. 2, each protein in our proposed approach is treated as a set of terms (document), where gene annotation terms are words. In order to construct the feature vector, the annotated GO terms of the genes related to each disease in Table 1 are extracted from the GO annotation database, then a set of terms is created for each category.

**Table 1:** List of diseases and number of known genes for each of them that have been used in experiments

Disease	Number of genes
Pancreatitis	6
Parkinson’s disease	21
Celiac disease	16
Atherosclerosis	43
Esophageal cancer	8
Crohn’s disease	17
Breast cancer	27
Alzheimer’s disease	21
Ulcerative colitis	24
Endometriosis	5
Cirrhosis	7
Myocardial infarction	32
Tuberculosis	12
Lymphoma	7
Rheumatoid arthritis	24
Asthma	29

Suppose the sets $MF\_termset\left({G}_{i}\right)=\left\{{F}_{1},{F}_{2}, \dots , {F}_{l}\right\}$, $CC\_termset\left({G}_{i}\right)=\left\{{C}_{1},{C}_{2}, \dots , {C}_{q}\right\}$, and $BP\_termset\left({G}_{i}\right)=\left\{{P}_{1},{P}_{2}, \dots , {P}_{r}\right\}$ correspond to the Molecular Function, Cellular Component terms, and Biological Process terms of the gene ${G}_{i}$, respectively. After extracting these terms, the three sets of terms,${G}_{MF}= {\bigcup }_{\forall i}MF\_termset\left({G}_{i}\right)$, ${G}_{CC}= {\bigcup }_{\forall i}CC\_termset\left({G}_{i}\right)$, and ${G}_{BP}= {\bigcup }_{\forall i}BP\_termset\left({G}_{i}\right)$, are generated for the genes associated with the diseases in Table 1. Then, using each of these sets, three feature maps are created as follows:

2 $$G_{MF} = \left\{ {F_{1} ,F_{2} ,F_{3} , \ldots ,F_{m} } \right\}$$

3 $$G_{CC} = \left\{ {C_{1} ,C_{2} ,C_{3} , \ldots ,C_{m} } \right\}$$

4 $$G_{BP} = \left\{ {P_{1} ,P_{2} ,P_{3} , \ldots ,P_{m} } \right\}$$

Finally, for each gene ${G}_{i}$, depending on the presence or absence of each GO term in the ${G}_{MF}$, ${G}_{CC},$ and ${G}_{BP}$ sets in the term sets of ${G}_{i}$, three feature vectors with equal length are constructed according to Eqs. (5)–(7).

5 $$F_{{G_{i} }} = \left\{ {F_{i,1} ,F_{i,2} ,F_{i,3} , \ldots ,F_{i,m} } \right\}, F_{i,j} = \left\{ {\begin{array}{*{20}l} 1 \hfill & {if\;F_{j} \in MF\_termset \left( {G_{i} } \right)} \hfill \\ 0 \hfill & {else} \hfill \\ \end{array} } \right.$$

6 $$C_{{G_{i} }} = \left\{ {C_{i,1} ,C_{i,2} ,C_{i,3} , \ldots ,C_{i,m} } \right\}, \;C_{i,j} = \left\{ {\begin{array}{*{20}l} 1 \hfill & {if\;C_{j} \in CC\_termset \left( {G_{i} } \right)} \hfill \\ 0 \hfill & {else} \hfill \\ \end{array} } \right.$$

7 $$P_{{G_{i} }} = \left\{ {P_{i,1} ,P_{i,2} ,P_{i,3} , \ldots ,P_{i,m} } \right\},\;\; P_{i,j} = \left\{ {\begin{array}{*{20}l} 1 \hfill & {if\;P_{j} \in PF\_termset \left( {G_{i} } \right)} \hfill \\ 0 \hfill & {else} \hfill \\ \end{array} } \right.$$

where ${F}_{{G}_{i}}$, ${C}_{{G}_{i}}$ and ${P}_{{G}_{i}}$ are feature vectors constructed based on the Molecular Function, Cellular Component, and Biological Process terms for the ${G}_{i}$, respectively.

### Convolutions on graph

In this paper, we investigate a multi-layer graph convolutional network (GCN) that operates according to the following propagation rule [38]:

8 $$H^{{\left( {i + 1} \right)}} = \sigma \left( {W^{i} \tilde{A}H^{i} } \right)$$

where $\widetilde{A }=A+{I}_{N}$ is the adjacency matrix of the undirected graph $G$ with added self-connections. ${I}_{N}$ indicates the identity matrix, and ${W}^{i}$ is a layer-specific trainable weight matrix. $\sigma \left(.\right)$ denotes an activation function, such as the $ReLU\left(.\right)=\mathrm{max}\left(0,.\right).$ ${H}^{(i)} \in {R}^{N\times D}$ represents the matrix of activations in the ${i}^{th}$ layer; ${H}^{0}=X.$ $X$ is the matrix of node (gene) feature vectors that is constructed in the previous step. Since the features have values of either 0 or 1, the multiplication operator uses the OR operation instead of the addition operation, and the multiplication of the two matrices is rewritten as follows:

9 $$\begin{aligned} & C = A*H \\ & C = \left[ {\begin{array}{*{20}c} {c_{11} } & \cdots & {c_{1n} } \\ \vdots & \ddots & \vdots \\ {c_{n1} } & \cdots & {c_{nn} } \\ \end{array} } \right],\;\; A = \left[ {\begin{array}{*{20}c} {a_{11} } & \cdots & {a_{1n} } \\ \vdots & \ddots & \vdots \\ {a_{n1} } & \cdots & {a_{nn} } \\ \end{array} } \right],\;\;{\text{and}}\;\; H = \left[ {\begin{array}{*{20}c} {h_{11} } & \cdots & {h_{1m} } \\ \vdots & \ddots & \vdots \\ {h_{n1} } & \cdots & {h_{nm} } \\ \end{array} } \right] \\ & {\text{such}}\;{\text{that}} \\ & c_{ij} = \left\{ {\begin{array}{*{20}l} 1 \hfill & {if c_{ij}^{^{\prime}} \ge 1} \hfill \\ 0 \hfill & {else} \hfill \\ \end{array} } \right. \\ & {\text{where}}\;\;c_{ij}^{^{\prime}} = a_{i1} h_{1j} + a_{i2} h_{2j} + \cdots + a_{in} h_{nm} = \mathop \sum \limits_{k = 1}^{n} a_{ik} h_{km} ,\;for\;i = 1, \ldots ,n \;and\;j = 1, \ldots ,m \\ \end{aligned}$$

### Semi-supervised node classification

In this paper, we use a graph-based semi-supervised learning method presented in [8]. This method requires a graph adjacency matrix $A,$ labeled samples ${x}_{1:L},$ ${y}_{1:L}$, and unlabeled samples ${x}_{L+1:L+U}$ as inputs. There is an embedding indicated by ${e}_{i}$ for each sample $i$. This framework is developed using feed-forward neural networks. The $k-th$ hidden layer of the network for the input feature vector $x$ is formulated as:

10 $$h^{k} \left( x \right) = ReLU\left( {W^{k} h^{k - 1} \left( x \right){ } + { }b^{k} } \right)$$

where ${W}^{k}$ and ${b}^{k}$ indicate parameters of the $h-th$ layer and ${h}^{0}\left(x\right)=x$. In this study, $ReLU(x) = max(0, x)$ is used as the nonlinear function. The loss function can be formulated as follows:

11 $${\mathcal{L}}_{s} + { }\lambda {\mathcal{L}}_{u}$$

where ${\mathcal{L}}_{s}$ and ${\mathcal{L}}_{u}$ are supervised loss based on the labeled part of the graph, and unsupervised loss of predicting the graph context, respectively. The semi-supervised learning model is outlined in the following by forming an unsupervised loss based on sample context from the graph, and then the supervised loss.

### Unsupervised loss of predicting the graph context

In order to formulate unsupervised loss, a sampling context method has been used. In this method, $(i, c, \gamma )$ is sampled from a distribution, where $i$ indicates a sample and $c$ is a context if $\gamma = +1,$ then $(i, c)$ is a positive pair, and if $\gamma = -1,$ then it is a negative pair. Based on $(i, c, \gamma )$, the cross-entropy loss is minimized by classifying $(i, c)$ to a binary label $\gamma$:

12 $$- {\mathbb{I}}\left( {\gamma = 1} \right) log \sigma \left( {w_{c}^{T} e_{i} } \right) - I\left( {\gamma = - 1} \right) log \sigma \left( { - w_{c}^{T} e_{i} } \right) ,$$

where $\sigma$ is the sigmoid function formulated as$\sigma (x) = 1/(1 +{ e }^{-x} )$, and ${\mathbb{I}}(.)$ is an indicator function that outputs $1$ when the argument is true; otherwise,$0$. As a result, the unsupervised loss is as follows:

13 $${\mathcal{L}}_{u} = - {\mathbb{E}}_{{\left( {i, c, \gamma } \right)}} \log \sigma \left( {\gamma w_{c}^{T} e_{i} } \right)$$

The distribution $p(i,c,\gamma )$ can be directly derived from the sampling methodology. This method samples two types of context. The first type of context uses graph $\mathrm{A}$ to encode the structure (distributional) information, and the second type uses labels to inject label information into the embeddings.

### Supervised loss

In order to formulate supervised loss, an embedding $e$ is defined for each sample. The embedding $e$ is formed by applying ${l}_{1}$ layers on the input feature vector$, e = {h}^{{l}_{1}}(x)$. Then, other remaining ${l}_{2}$ layers are applied on the $:$ ${h}^{{l}_{2}}\left(e\right)={h}^{{l}_{2}}\left({h}^{{l}_{1}}(x)\right)$, represented as ${h }^{l}(x)$ where$l ={ l}_{1} + {l}_{2}$. Based on this formulation, embedding $e$ is a parameterized function of the feature$x$. In more detail, the probability of predicting label y is expressed as follows:

14 $$p(y|x) = \frac{{{\text{exp}}\left[ {h^{k} \left( x \right)^{T} , h^{l} \left( x \right)^{T} } \right]w_{y} }}{{\mathop \sum \nolimits_{{y^{\prime}}} {\text{exp}}\left[ {h^{k} \left( x \right)^{T} , h^{l} \left( x \right)^{T} } \right]w_{{y^{\prime}}} }}$$

15 $${\mathcal{L}}_{s} = - \frac{1}{L} \mathop \sum \limits_{i = 1}^{L} \log p\left( {y_{i} {|}x_{i} } \right)$$

where ${h }^{k}(x)$ is obtained by applying $k$ layers on the input feature vector $x$. When ${e}_{i}$ replaced with ${h}^{{l}_{1}}\left({x}_{i}\right)$ in Eq. (13), the loss function is:

16 $$- \frac{1}{L} \mathop \sum \limits_{i = 1}^{L} \log p\left( {y_{i} {|}x_{i} } \right) - \lambda {\mathbb{E}}_{{\left( {i, c, \gamma } \right)}} \log \sigma \left( {\gamma w_{c}^{T} h^{{l_{1} }} \left( {x_{i} } \right)} \right)$$

**Fig. 3:**
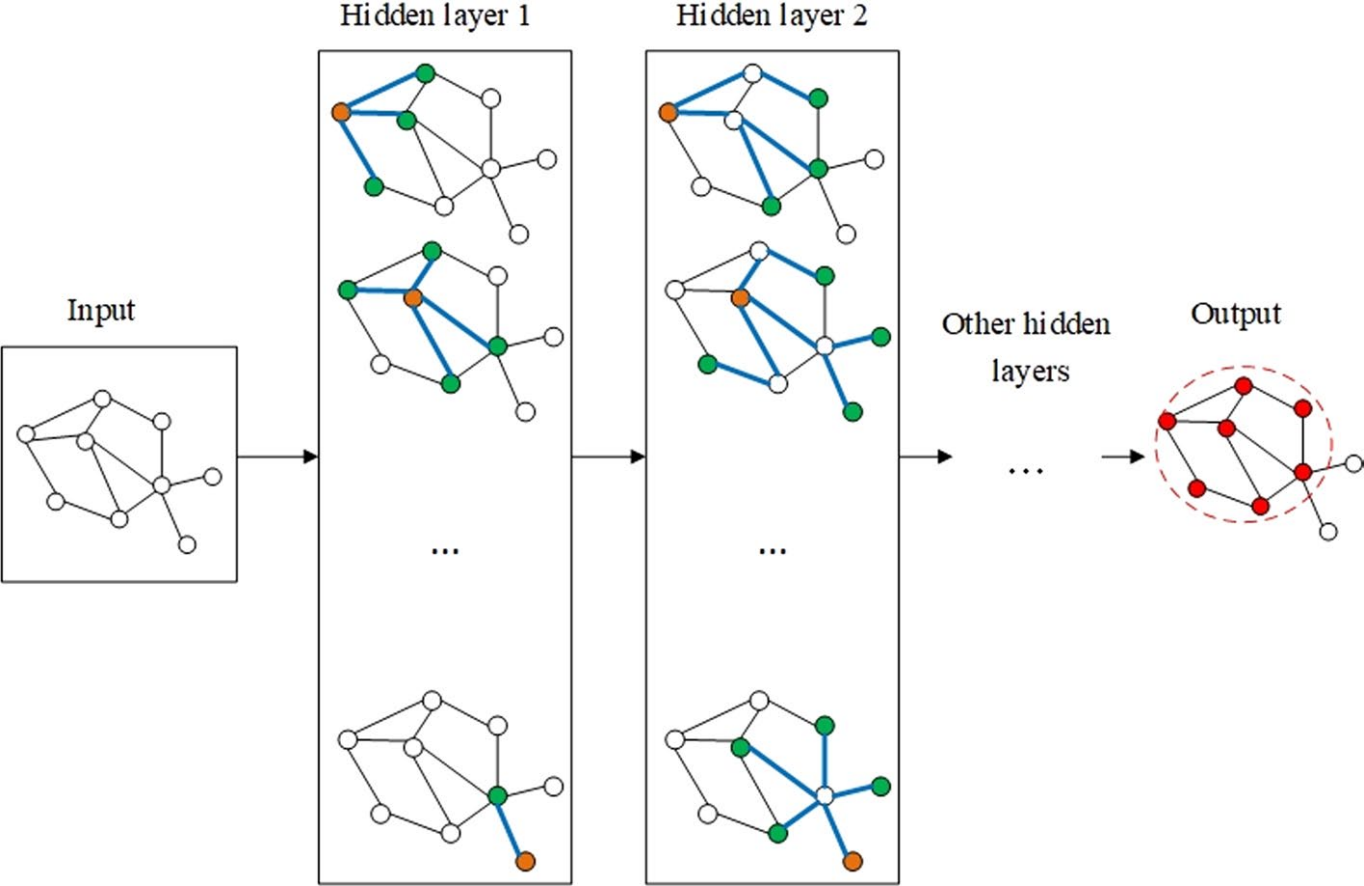
The overall view of the proposed graph convolutional network method

Figure 3 illustrates a schematic representation of the overall model for semi-supervised learning, using a multi-layer GCN.

### Training

After sampling a batch of labeled samples, we optimize the loss function of class label prediction by performing a gradient step. Following that, to optimize the loss function of context prediction, a batch of context $(i, c, \gamma$) is sampled, and another gradient step is performed. The procedure of the training model is presented in Algorithm 1 [8].
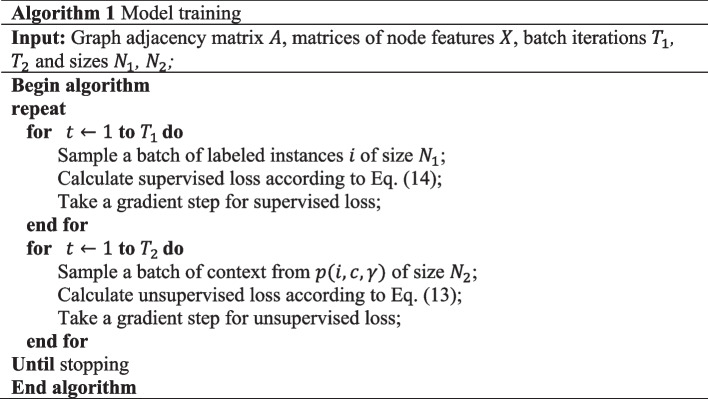


### Stochastic gradient descent

Our model is trained using stochastic gradient descent (SGD) [54]. In this method the excess error $\mathcal{E}={\mathbb{E}} [ E\left({\widetilde{f}}_{n}\right)-E({f}^{*}) ]$ can be divided into three parts according to this method:

17 $${\mathcal{E}} = {\mathcal{E}}_{app} + {\mathcal{E}}_{est} + {\mathcal{E}}_{opt}$$

where ${\mathcal{E}}_{app}$ represents the approximation error which is a measure of how closely functions in $\mathcal{F}$ can approximate the optimal solution ${f}^{*}$. A larger family of functions can reduce the approximation error. ${\mathcal{E}}_{app}$ is calculated using the following equation:

18 $${\mathcal{E}}_{app} = {\mathbb{E}}\left[ {E\left( {f_{{\mathcal{F}}}^{*} } \right) - E\left( {f^{*} } \right)} \right]$$

In this case, ${f}^{*}= {argmin}_{f} E(f)$ represents the best possible prediction function, $\mathcal{F}$ is a parametrized family of functions, and ${f}_{\mathcal{F}}^{*}= {argmin}_{f\in \mathcal{F}} E(f)$ represents the best function in $\mathcal{F}$.

${\mathcal{E}}_{est}$ is the estimation error which used to determine how well we can minimize the empirical risk ${E}_{n}(f)$ instead of the expected risk $E(f)$. Smaller families of functions or larger training sets can reduce the estimation error. The following equation is used to calculate ${\mathcal{E}}_{est}$:

19 $${\mathcal{E}}_{est} = {\mathbb{E}}\left[ {E\left( {f_{{\mathcal{F}}}^{*} } \right) - E\left( {f^{*} } \right)} \right]$$

where ${f}_{n}= {argmin}_{f\in \mathcal{F}} {E}_{n}(f)$ is the empirical optimum.

${\mathcal{E}}_{opt}$ is the optimization error that measures how the approximate optimization affects the expected risk. By running the optimizer longer, you can reduce the optimization error. Depending on the training set size and the family of functions, additional computing time may be required. ${\mathcal{E}}_{opt}$ is calculated as follows:

20 $${\mathcal{E}}_{opt} = {\mathbb{E}}\left[ {E\left( {\tilde{f}_{n} } \right) - E\left( {f_{n} } \right)} \right]$$

where ${\widetilde{f}}_{n}$ is a solution that minimizes the objective function with a predetermined accuracy ${E}_{n}\left({\widetilde{f}}_{n}\right)<{E}_{n}\left({f}_{n}\right)+\rho$.

The gradient descent can be represented as follows:

21 $$\mathop {\min }\limits_{{{\mathcal{F}}, \rho ,n }} {\mathcal{E}} = {\mathcal{E}_{app}} + {\mathcal{E}_{est}} + {\mathcal{E}_{opt}} \;\;subject\;to \left\{ {\begin{array}{*{20}c} {\mathcal{n} \le \mathcal{n}_{\max } } \\ {T\left( {{\mathcal{F}}, \rho ,\mathcal{n}} \right) \le T_{\max } } \\ \end{array} } \right.$$

where ${T}_{max}$, and ${\mathcal{n}}_{max}$ are maximal computation time and the maximal training set size, respectively. $\rho$ is the optimization accuracy, and $\mathcal{n}$ represents the number of examples.

### Candidate gene prioritization

In order to rank candidate genes, we used the output from the model, where the output with the higher value corresponds to the higher ranking. A final ranking is computed by averaging the results obtained from all three types of constructed feature vectors for genes. The procedure of the proposed candidate gene prioritization is represented in Algorithm 2.
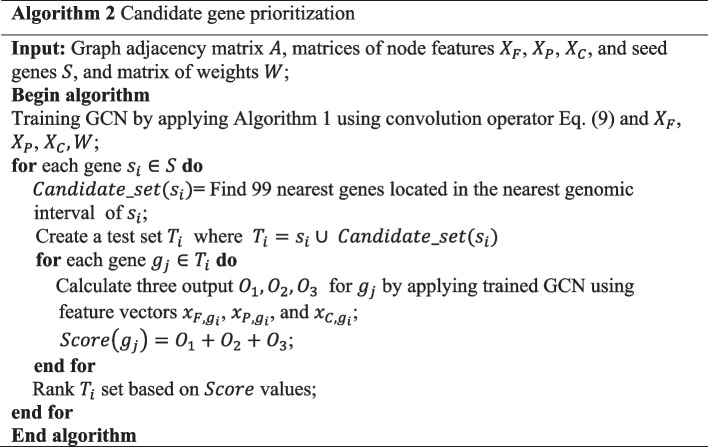


### Computational complexity analysis

A computational complexity analysis is performed in this section. In the first step, three feature vectors were constructed for each gene. The computational complexity of this step is $O(n)$ where $n$ is the number of genes in the original set. In the second step, a graph convolutional network is trained using the semi-supervised learning approach. The computational complexity of the forward convolution is $O(L.I.\mathrm{n}.{m}^{2})$ where $L$ is the number of layers, $I$ represents the maximum number of iterations, and $m$ is the number of features. Moreover, the computational complexity of the semi-supervised training is $O(L.I.({N}_{1}+{N}_{2}).{m}^{2})$ where ${N}_{1}$, and ${N}_{2}$ are the size of a batch of labeled instances and a batch of context from $p(i,c,\gamma )$, respectively. Therefore, the computational complexity of this step of the proposed method is $O(L.I.\mathrm{n}.{m}^{2}+L.I.({N}_{1}+{N}_{2}).{m}^{2})$, which can be reduced to $(L.I.\mathrm{n}.{m}^{2})$. According to the computational complexities of all steps, the final computational complexity of the proposed method will be $O(n+L.I.\mathrm{n}.{m}^{2})$ which is reduced to $O(L.I.\mathrm{n}.{m}^{2})$.

## Results

In this section, several experiments are conducted to evaluate the performance of the proposed method. TensorFlow [55] is used in practice for an efficient implementation of Eq. (8) on GPUs with sparse-dense matrix multiplications.

### Dataset

Our experiments utilize human protein–protein interaction (PPI) data retrieved from NCI's Entrez Gene Database [56]. Interaction data on this database is sourced from other databases. For example, HPRD, Biological General Repository for Interaction Datasets (BioGRID), and Biomolecular Interaction Network Database (BIND) are among the sources. The final PPI network has 8959 proteins and 33528 interactions between these proteins after removing nodes with no interactions. In this study, GPL10558 ontology data was used to construct the feature vectors for each gene, which contains 44945 terms. To construct the feature vector from this collection, 1671 MF terms, 1653 CC terms, and 1644 BP terms were extracted for 299 genes known for diseases, and then three feature vectors were created for each gene based on these terms. In all three feature vectors, the length equals the number of terms in the largest term set, which is 1671.

### Experimental setting

We train a four-layer GCN and evaluate its performance on a test set. A test set of 100 genes containing a disease gene from a set of genes associated with a disease is constructed, along with 99 genes located in the nearest genomic interval. Additional experiments are performed using deeper models with layers up to 10. Training all models for a maximum of 200 epochs (training iterations) is performed using stochastic gradient descent (SGD), with a learning rate of 0.01 and early stopping at the window size of 10, which means that the training stops after a validation loss does not decrease for 10 consecutive iterations. Using the method described in [57], we initialize the weights.

Using the evaluation criteria described following, we evaluate the quality of each algorithm's ranking. Leave-one-out cross-validation approach is used to assess the effectiveness of different methods for prioritizing genes associated with the disease. Based on the used dataset, we run the steps described below for every gene $u$ known to cause disease D:

- In this study, the target gene $u$ is placed in a sophisticated linkage interval along with 99 other genes located closest to it in terms of genomic proximity. Candidate set C is made up of genes within this artificial linkage interval (including $u$).
- Genes in C are ranked by applying each prioritization algorithm.
- The gene $u$ is removed from the list of genes associated with D. During the experiment, $u$ is called the target gene. The rest of the gene set related to D form seed set S.

### Evaluation criteria

By varying the threshold on the rank of a gene to be classified as a "predicted disease gene", first ROC curves (precision/recall) are plotted. As a result of consideration of threshold ranks, sensitivity can be defined as the percentage of lists where the left-out gene is ranked higher than the threshold and specificity as the percentage of lists where the left-out gene is ranked lower than the threshold. Based on various threshold ranks, sensitivity and specificity values were calculated and used to construct a ROC curve, from which AUC was calculated. Gene prioritization methods are evaluated by using AUC as a standard measure of efficiency. If AUC = 100%, the defector gene is prioritized as the top priority. While AUC = 50% indicates a random ranking of the defector genes. The precision, recall, and F1-score calculations are also performed, as part of the evaluation of our method according to Eqs. (22)–(24).

22 $$p = Precision = \frac{TP}{{TP + FP}} \times 100$$

23 $$r = Recall = \frac{TN}{{TP + FP}} \times 100$$

24 $$F1 - score = \frac{{\left( {2*p*r} \right)}}{p + r} \times 100$$

where $TP,FP, and\, TN$ are respectively the numbers of true positive, false positive, and true negative.

Using the Genetic Association Database (GAD) [58], our analysis included 16 diseases and the known genes associated with each one. In Table 1, the number of known proteins associated with each disease used in this study is listed.

### Performance evaluation

The proposed method’s results are compared with eight state-of-the-art methods. Brief descriptions of these methods can be found in the following:

**GPEC** [22]: GPEC is designed for recognizing genes that are more likely associated with particular diseases. An algorithm based on random walk with restart along with a gene-protein link network is used in this plugin to identify gene priorities.

**DADA** [59]: Different uniform prioritization methods are effectively combined with statistical regulation strategies in this method. PPI network is used to assess the degree distribution of known diseases and candidate genes using several statistical adjustment methods.

**HSSVM** [60]: Using the HeteSim [61] measure, this method calculates the relative importance of different or the same type of node types in a heterogeneous network. HSSVM combines the HeteSim measure with a machine learning method to account how similar nodes are. Each path contributes differently to the relevance score, so the machine learning method is utilized to determine the weight for each path. In order to determine the weights of various paths, a positive and unlabeled learning method was implemented.

**Arete** [62]: In this framework, two existing network-based gene prioritization methods are combined using an isolation forest-based integrative ranking method. The method includes a random walk with restart (RWR) and an iterative neighborhood-based approach.

**WCR**_**STAR**_ [63]: To prioritize disease genes, this method integrates tissue-specific molecular networks. As a result, each disease can have its own tissue-specific network(s). Based on network propagation, this method formulates candidate gene prioritization as an optimization problem. If a disease has multiple tissue-specific networks, based on this method, each tissue-specific network can be assessed for its relative importance. It can deal with noisy and incomplete network data. The optimization problem is solved by creating fast algorithms whose time complexity increases linearly with the number of nodes in the molecular networks.

**C-PUGP** [64]: In this method, positive-unlabeled (PU) learning technique is utilized for gene prioritization using clustering and one-class algorithms. In this method, it has been attempted to make a set of reliable negative examples in three steps. First, positive samples are clustered. In the next step, a single-class sorting algorithm is taught using the clusters obtained from the previous step, and in the last step, a dependable negative sample is defined as the convergent point of negative data.

**TLGP** [65]: A transfer learning-based gene prioritization (TLGP) is proposed in this method, using knowledge transferred from other cancers (source) to prioritize genes in cancer (target). This method is based on the hypothesis that knowledge sharing between cancers improves the performance of gene prioritization.

**GPrior** [66]: A tool based on positive-unlabeled learning that selects an optimal set of classification algorithms including, logistic regression, support vector machine (SVM), random forest, decision tree, and adaptive boosting to tune the proposed method for each particular phenotype.

**Fig. 4:**
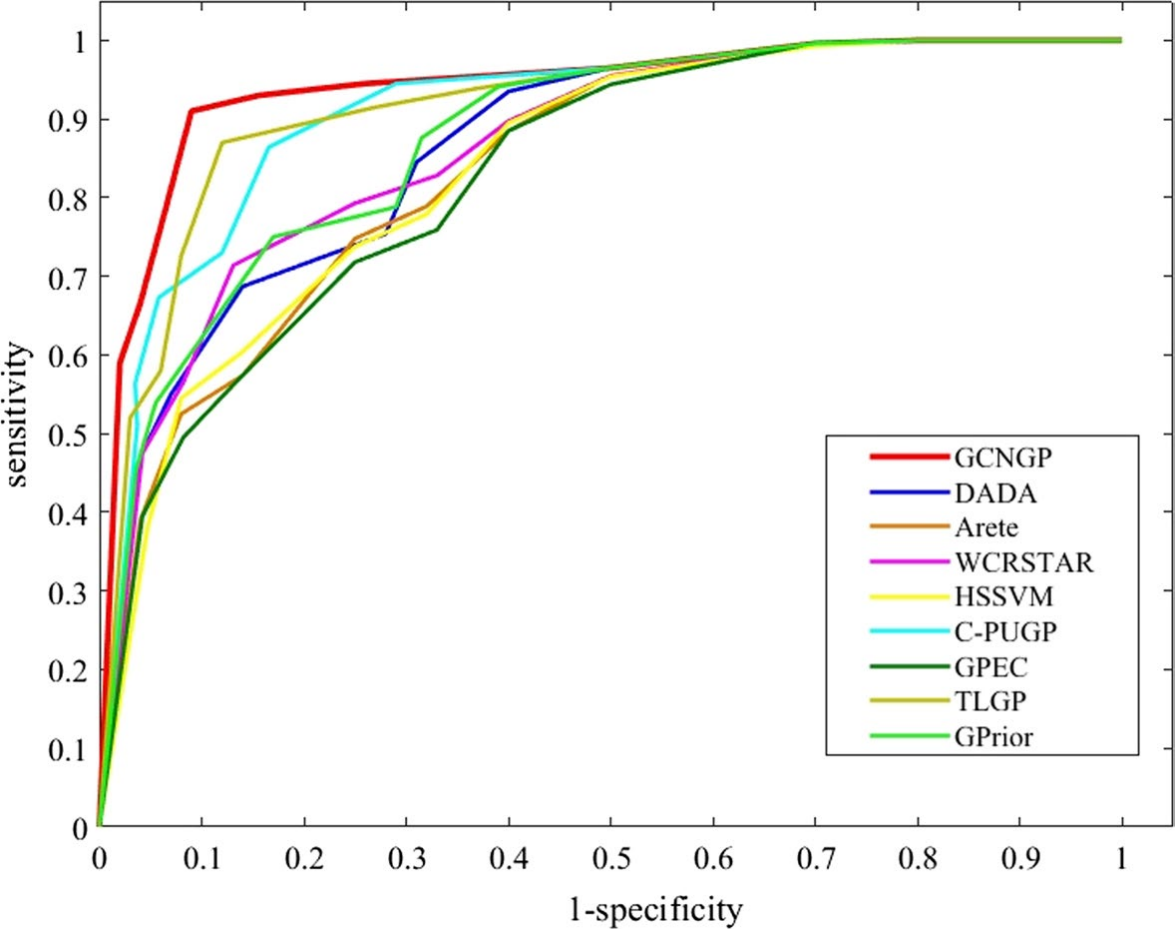
ROC curves corresponding to all the disease genes for GCNGP and other methods

**Fig. 5:**
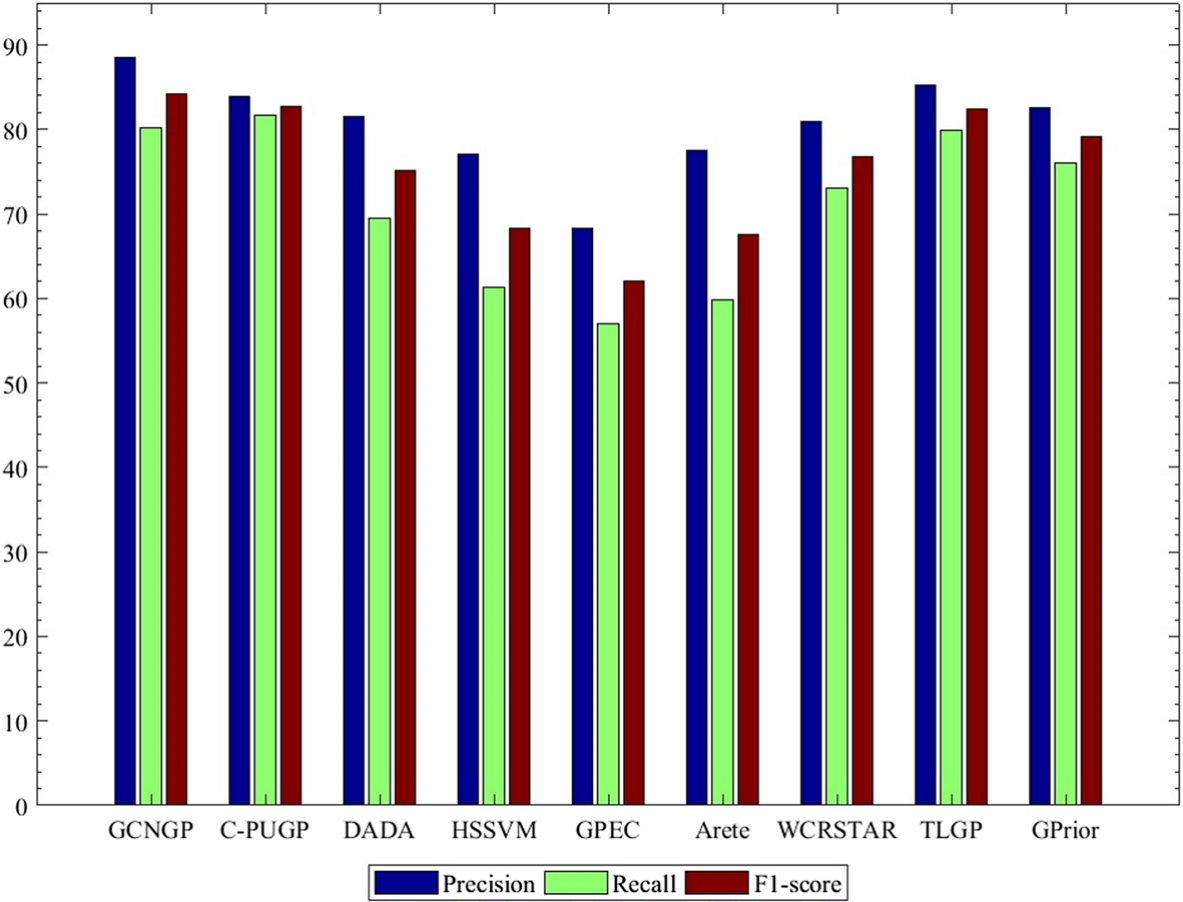
Comparison between GCNGP and other methods based on precision, recall, and F1-score

In order to impartial assessment, the same training and test sets and leave-one-out cross-validation for all of the eight methods above are used. The comparison of the results is presented in Tables 2, 3, and Figs. 4, 5. Tables 2, and 3 show the AUCs calculated for each of 16 diseases using our method compared to eight other methods for thresholds equal to 5, and 10, respectively. The results show that the proposed method often has higher AUCs values than other methods in each case. The increase of average AUCs value is 3.4 and 3.56 percent better than the best method, the TLGP method, for thresholds equal to 5 and 10, respectively.

**Table 2:** The AUCs values of different methods over 16 diseases for threshold = 5

Disease	Methods								
	GCNGP	C-PUGP	DADA	HSSVM	GPEC	Arete	WCR_STAR_	TLGP	GPrior
Pancreatitis	80.76	76.95	71.99	68.51	50.72	67.55	72.26	77.12	74.71
Parkinson’s disease	75.28	69.72	61.45	57.38	48.65	51.39	68.48	71.86	68.95
Celiac disease	77.95	71.07	67.61	61.27	42.96	53.02	72.28	73.90	70.43
Atherosclerosis	80.25	78.81	73.66	70.33	61.48	64.14	75.80	78.09	75.24
Esophageal cancer	78.61	76.15	69.63	65.95	60.52	63.38	69.92	74.55	69.78
Crohn’s disease	69.90	66.39	62.05	59.89	52.66	60.16	66.21	66.98	64.36
Breast cancer	74.08	71.22	68.76	68.29	55.79	61.05	69.54	72.57	69.19
Alzheimer’s disease	76.39	69.76	66.18	61.90	59.37	67.80	68.17	71.83	68.42
Ulcerative colitis	67.83	61.98	59.03	57.64	49.13	59.74	62.97	63.81	62.85
Endometriosis	84.30	80.59	74.84	70.61	53.85	77.32	79.53	80.87	79.66
Cirrhosis	59.92	55.09	51.76	49.47	41.62	52.97	56.88	55.69	54.18
Myocardial Infarction	71.48	65.83	60.91	56.17	44.21	55.82	66.02	67.71	64.13
Tuberculosis	78.75	74.07	67.22	66.19	52.98	67.46	71.44	75.05	73.22
Lymphoma	73.87	71.65	58.11	55.72	50.78	54.29	72.68	71.93	71.07
Rheumatoid Arthritis	66.09	62.53	61.68	59.16	55.73	55.89	61.95	62.64	61.16
Asthma	63.94	59.86	54.82	55.27	53.04	57.98	58.85	60.28	58.39
Average	73.71	69.48	64.36	61.48	52.09	60.62	68.31	70.31	67.86

**Table 3:** The AUCs values of different methods over 16 diseases for threshold = 10

Disease	Methods								
	GCNGP	C-PUGP	DADA	HSSVM	GPEC	Arete	WCR_STAR_	TLGP	GPrior
Pancreatitis	93.65	87.58	85.19	79.24	66.86	72.39	81.10	90.01	86.25
Parkinson’s disease	88.82	76.84	69.98	68.84	60.92	66.48	77.66	83.65	75.19
Celiac disease	87.39	79.75	74.25	70.15	59.28	69.36	80.85	79.17	71.92
Atherosclerosis	96.55	91.66	90.08	81.95	72.67	79.14	89.28	92.40	90.38
Esophageal cancer	87.44	86.78	81.63	78.11	71.09	76.38	78.34	85.82	82.59
Crohn’s disease	82.73	80.25	77.29	76.85	69.16	76.50	79.09	80.07	78.44
Breast cancer	87.66	85.62	80.76	75.70	70.05	73.81	81.54	86.38	82.11
Alzheimer’s disease	90.45	82.19	83.64	82.96	76.27	80.25	84.17	85.87	81.95
Ulcerative colitis	79.36	78.46	77.96	71.27	69.91	72.74	75.52	77.98	75.16
Endometriosis	96.05	90.44	88.32	85.50	70.85	81.65	87.76	91.16	89.18
Cirrhosis	74.48	70.91	71.80	66.67	59.09	68.04	71.45	71.43	70.62
Myocardial infarction	91.69	87.35	87.95	82.41	68.66	75.90	85.69	89.05	86.57
Tuberculosis	95.35	91.80	89.15	80.29	71.18	79.82	90.13	92.34	90.48
Lymphoma	92.81	88.39	85.78	82.33	73.06	82.66	89.92	89.15	86.39
Rheumatoid arthritis	84.97	80.16	80.72	72.54	70.33	71.68	79.35	80.93	78.69
Asthma	86.19	83.92	81.18	79.46	64.47	77.49	80.97	83.11	81.27
Average	88.47	83.88	81.61	77.14	68.37	75.27	82.05	84.91	81.70

There are 299 genes associated with all 16 experimental diseases. For each gene associated with a disease, we generated a list of 100 test genes according to their rank. We constructed a ROC curve based on all 299 rank lists, which can be seen in Fig. 4. Because the ROC curve for our method is above other methods, the AUC value of our method is larger than other methods. Figure 5 confirms that our proposed method is the best in terms of precision and F1-score, while C-PUGP is better in terms of recall. It may be due to its focus on the production of suitable negative samples that C-PUGP performed better in recall. The increase in performance is 3.6 and 1.5 percent better than best method, C-PUGP method, in terms of precision, and F1-score, respectively.

**Fig. 6:**
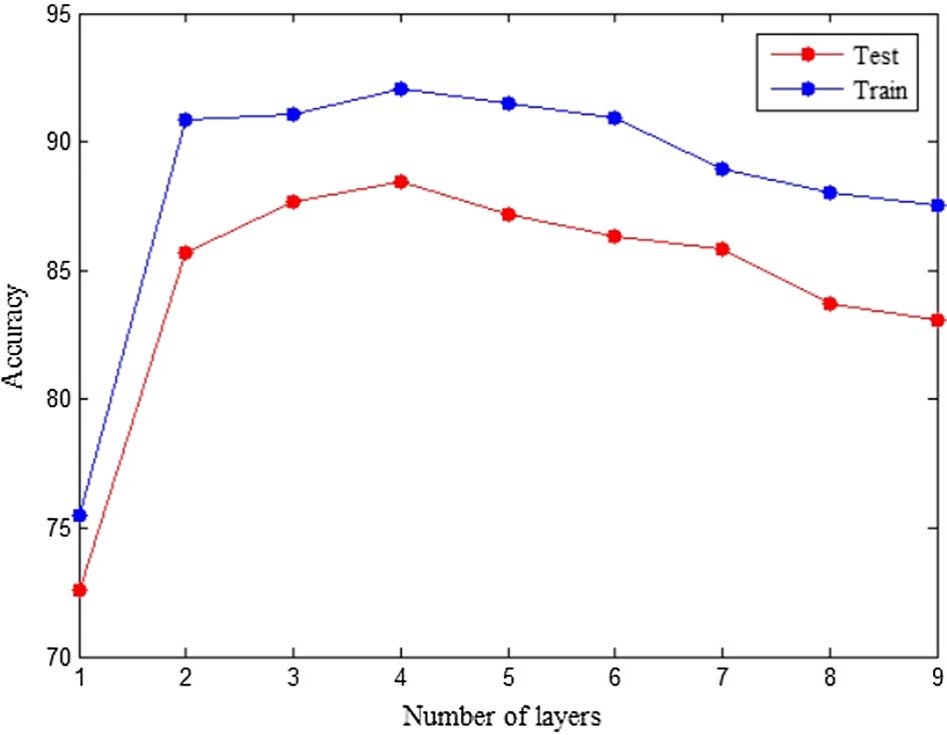
A comparison of classification accuracy for the different number of layers. Each marker indicates the average classification accuracy for the training and testing phases

The best results are obtained for the datasets considered here by using a three- or four-layer model. As layers deeper than 7 are added, training without residual connections becomes increasingly difficult, as the number of nodes in a model's context increases in proportion to the size of their $l$ th-order neighborhood. A growing model depth can also cause overfitting due to the increased number of parameters. Classification performance is influenced by the depth (number of layers) of a proposed model shown in Fig. 6.

Furthermore, the proposed method has been compared to graph convolutional networks based methods. These methods are described briefly below:

**PGCN** [41]: This method employs graph convolutional neural networks to learn embedding for genes and diseases. In this method a heterogeneous network is constructed by putting together the genetic interaction network, the human disease similarity network, and the disease-gene association network. Gene prioritization is treated as a link prediction problem in this method.

**RGCN** [42]: As part of RGCN, disease similarities, gene similarities, and disease-gene associations are used to construct a multi-relational network. Link prediction is used here to model the disease gene prioritization problem.

**GCAS** [43]: The GCAS method infers new phenotype-gene associations from this initial set of associations using graph convolution. Genes, diseases, phenotypes, pathways, and ontological associations are integrated into a heterogeneous network in this method.

**GCN-MF** [44]: GCN-MF combines Graph Convolutional Networks (GCN) with matrix factorization in order to solve the problem of disease-gene association. To reduce the effect of sparsity, a margin control loss function is used, in this method.

**Fig. 7:**
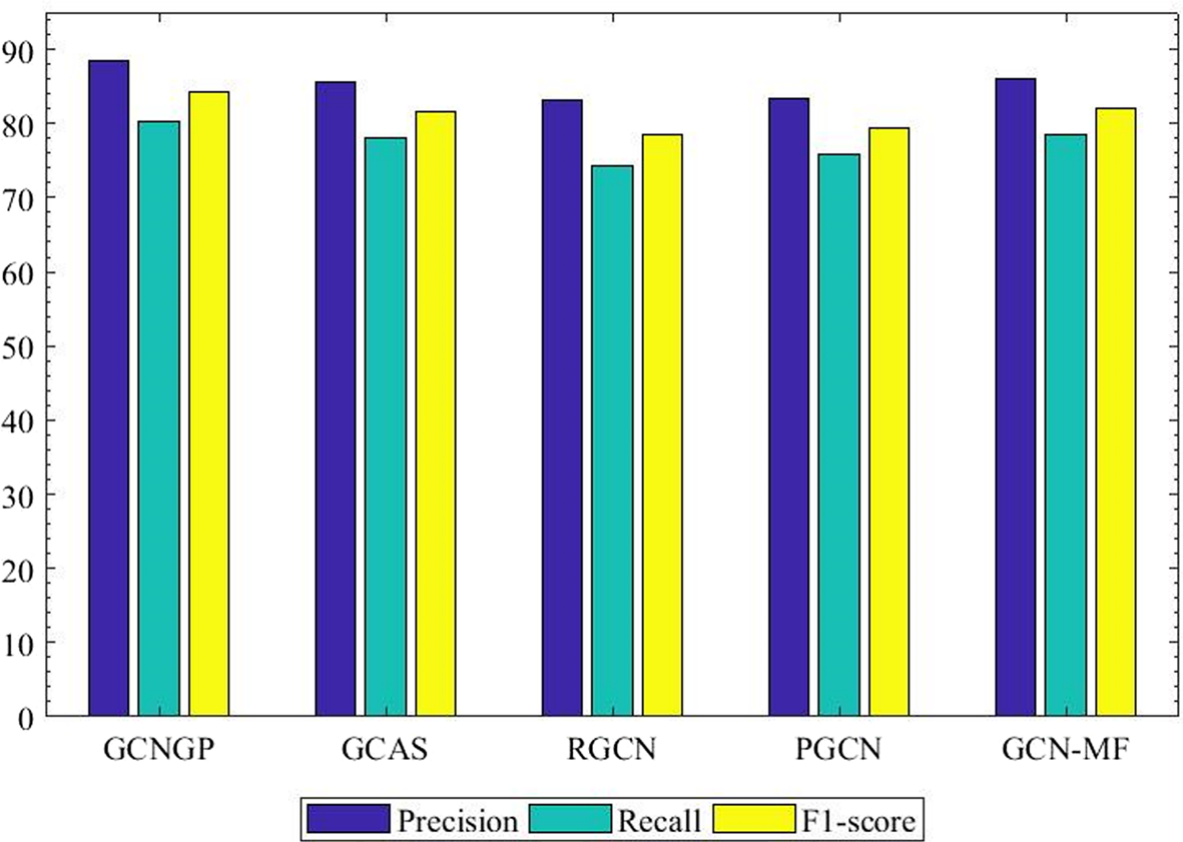
Comparison between GCNGP and other graph convolutional networks methods based on precision, recall, and F1-score

Tables 4 and 5 represent the AUCs calculated for each of 16 diseases using our method compared to four other methods for threshold equal to 5, and 10, respectively. These results demonstrate that our proposed method not only outperforms classical models, but it has superiority in comparison to the recently graph convolutional networks developed models. Figure 7 shows the results of our proposed method compared to other graph convolutional networks methods in terms of precision, recall, and F1-score.

**Table 4:** The AUCs values of different graph convolutional networks methods over 16 diseases for threshold = 5

Disease	Methods				
	GCNGP	GCAS	RGCN	PGCN	GCN-MF
Pancreatitis	80.76	73.38	76.18	78.01	78.57
Parkinson’s disease	75.28	71.25	70.90	74.59	73.16
Celiac disease	77.95	73.81	70.39	71.27	75.82
Atherosclerosis	80.25	76.66	73.11	75.05	75.98
Esophageal cancer	78.61	75.15	74.77	75.86	79.10
Crohn’s disease	69.90	65.49	63.26	65.07	67.44
Breast cancer	74.08	71.69	71.87	70.78	72.88
Alzheimer’s disease	76.39	73.94	70.85	69.97	74.29
Ulcerative colitis	67.83	65.81	62.19	62.92	64.66
Endometriosis	84.30	81.17	79.67	79.95	83.13
Cirrhosis	59.92	56.94	55.09	56.19	57.75
Myocardial infarction	71.48	70.18	67.75	66.54	69.49
Tuberculosis	78.75	75.97	71.54	70.72	76.91
Lymphoma	73.87	72.45	69.19	70.04	71.97
Rheumatoid arthritis	66.09	64.93	63.17	61.59	64.68
Asthma	63.94	60.74	59.13	59.91	61.39
Average	73.71	70.60	68.69	69.28	71.70

**Table 5:** The AUCs values of different graph convolutional networks methods over 16 diseases for threshold = 10

Disease	Methods				
	GCNGP	GCAS	RGCN	PGCN	GCN-MF
Pancreatitis	93.65	90.44	88.18	89.05	89.25
Parkinson’s disease	88.82	87.29	84.75	83.69	85.90
Celiac disease	87.39	83.08	82.77	80.88	85.10
Atherosclerosis	96.55	92.97	89.35	91.64	94.89
Esophageal cancer	87.44	85.15	84.58	81.29	86.96
Crohn’s disease	82.73	81.36	78.84	80.01	80.52
Breast cancer	87.66	84.49	81.69	83.13	85.73
Alzheimer’s disease	90.45	85.25	83.86	82.49	88.27
Ulcerative colitis	79.36	75.77	72.61	74.16	77.07
Endometriosis	96.05	95.29	91.54	89.99	92.43
Cirrhosis	74.48	71.01	69.37	70.71	72.55
Myocardial infarction	91.69	86.82	82.95	85.36	89.18
Tuberculosis	95.35	94.66	91.62	92.13	93.69
Lymphoma	92.81	88.91	86.25	85.79	90.14
Rheumatoid arthritis	84.97	83.54	80.08	81.22	82.25
Asthma	86.19	83.09	82.92	83.88	84.24
Average	88.47	85.57	83.21	83.46	86.14

### Statistical analysis of the proposed method

We conduct the Friedman test [67] as a means of analyzing the performance of our method and those of the others. It is a nonparametric statistic used to evaluate the results of various methods on a range of various datasets. As a result, the rank of every gene prioritization method is determined based on the specific disease. The SPSS statistics acquired by IBM [68] are used for this purpose. Hypothesis $\mathrm{H}0$ is based on the similarity of the average ranks between the groups in the Friedman test. The null hypothesis is rejected if there is a significant difference between at least two groups. If the level of significance of the Friedman test is less than the level of error, it is impossible to determine whether the difference between at least two specimens is deducted from the results. To satisfy the constraint, the level of significance must be less than 0.05 since the test error is considered 5%. The Friedman test results of the proposed method are shown in Tables 6 and 7 in comparison to the other methods. Specifically, Table 6 lists the average rankings of all models based on different thresholds. Based on these results, it appears that the proposed method has the best ranking as compared with all other models. Based on all evaluated thresholds, it is possible to conclude that the GCNGP is the best performing method. Based on Table 7, we can see that the Friedman test returns a $P$ value of $3.13\times {10}^{-22}$ for threshold = 5. This value is less than 0.05, so it can be stated that the proposed method yields significantly different results. Further $P$ value from the Friedman test for threshold = 10 confirms that our method differs significantly from others.

**Table 6:** Average ranks of different methods for different threshold values

Threshold	Methods									
	GCNGP	C-PUGP	DADA		HSSVM	GPEC	Arete	WCR_STAR_	TLGP	GPrior
5	1	3.25		6.37	7.37	9	7.18	3.87	2.25	4.68
10	1	3.31		4.75	7	9	7.81	4.5625	2.56	4.93

**Table 7:** The results of the statistical test

	Threshold	
	5	10
Chi-Square	120.12	113.52
df	8	8
*P* value	$3.13\times {10}^{-22}$	$7.19 \times {10}^{-21}$

The Friedman test has also been performed for the proposed method compared to other graph convolutional networks methods. In Table 8, all graph convolutional networks models are ranked according to various thresholds. Compared to all other GCN-based models, the proposed method shows the best ranking. The Friedman test returns a P value of $3.79\times {10}^{-10}$ for threshold = 5 as shown in Table 9. This value is less than 0.05, so it can be stated that the proposed method yields significantly different results. Furthermore, our method is significantly different from other GCN-based methods based on the Friedman test for threshold = 10.

**Table 8:** Average ranks of different graph convolutional networks methods for different threshold values

Threshold	Methods					
	GCNGP	GCAS	RGCN		PGCN	GCN-MF
5	1.06	3		4.56	4.06	2.31
10	1	2.69		4.63	4.31	2.38

**Table 9:** The results of the statistical test for of different graph convolutional networks methods

	Threshold	
	5	10
Chi-square	49.9	56.65
*df*	4	4
*P* value	$3.79\times {10}^{-10}$	$1.5 \times {10}^{-11}$

## Discussion

Various factors contribute to the superior performance of the proposed GCNGP method over other compared gene prioritization methods. There are three contributions that help explain why the proposed method is superior.

1. One of the most problematic aspects of existing gene prioritization methods is the reliance on a single data resource. In order to overcome this problem, our proposed method takes advantage of different datasets. In our method, in addition to using the PPI network, the GO dataset was used to gather knowledge for each gene. For this purpose, three feature vectors were constructed using the GO dataset for each gene.
2. A majority of network-based methods rely solely on the intrinsic and structural properties of nodes. The proposed method also uses features based on other data sources in addition to the structural properties of each node, and in the end, a representation of each node is derived by applying the convolution of that node's features to its neighbors.
3. In some previous studies, the gene prioritization problem is viewed as a two-class classification, in which random unknown genes are depicted as negative examples. In this paper, the gene prioritization problem is considered a semi-supervised learning problem, that is trained and tested using the graph convolutional network, a popular graph deep learning models. Moreover, what distinguishes our method from others is the capability of propagating information in the PPI network as well as considering direct and indirect interactions between genes for ranking.

Memory limitations were the primary bottleneck in our proposed method due to the size of the feature vectors and the dimensions of the PPI network.

## Conclusion

A variety of machine learning approaches have been utilized to predict disease genes based on the rule that genes associated with the same disorder are more likely to have similar features; however, many of these methods still have shallow learning mechanisms. Furthermore, the lack of appropriate representations for genes is another challenge with disease gene identification. Such methods are focused on capturing the properties of genes. Additionally, traditional methods usually use disease genes as the positive training set and unknown genes as the negative training set to build binary classification models. There is no doubt that convolutional graph networks have a considerable amount of ability to learn graph representations and node classifications, and they have achieved excellent results for a variety of tasks and applications. This type of neural network architecture utilizes graph structure and incorporates aggregating information from neighbors in a convolutional way.

In this paper, we proposed a semi-supervised learning method based on a graph convolutional network to classify and rank candidate disease genes. We propose a novel method called GCNGP based on PPI network data, and an innovative method to create three feature vectors for genes based on the Molecular Function, Cellular Component and Biological Process terms from GO data. A customized convolutional network, and extracting the knowledge set from GO data and constructing feature vectors for each gene are the main idea and novelty of the proposed method. First, all Molecular Function, Cellular Component, and Biological Process terms of disease genes are extracted from the GO database. Then, for each gene three feature vectors are constructed. Second, a graph convolutional network is trained on PPIs networks and feature vectors of nodes from the previous step. Third, the validation of the proposed method is performed. The performance of proposed method is compared with eight well-known gene prioritization methods and four graph convolutional networks methods using a variety of criteria. The experimental results demonstrated that the proposed gene prioritization method can considerably outperform other methods. The use of edge features and network embedding methods such as node2vec could be a promising direction for future research. In addition to drug disease association and homolog detection for protein structure prediction, the framework we developed can be easily applied to other important problems in computational biology and biomedical network analysis.

## Data Availability

The datasets used in the present research can be accessed for free at http://www.ncbi.nlm.nih.gov and http://cbdm-01.zdv.uni-mainz.de/~mschaefer/hippie/index.php
